# *L*-Arginine Attenuates Heat Stress-Induced Oxidative Damage and Apoptosis in Bovine Neutrophils via NFE2L2-Mediated ROS Scavenging

**DOI:** 10.3390/vetsci13070628

**Published:** 2026-06-27

**Authors:** Shang Jiang, Haihua Feng, Chao Wang, Xiliang Du, Lin Lei, Wenwen Gao, Guowen Liu, Xinwei Li, Yuxiang Song

**Affiliations:** State Key Laboratory for Diagnosis and Treatment of Severe Zoonotic Infectious Diseases, Key Laboratory for Zoonosis Research of the Ministry of Education, Institute of Zoonosis, College of Veterinary Medicine, Jilin University, Changchun 130062, China; jiangshang2026@126.com (S.J.); fhh70@163.com (H.F.); wangchaojlu2026@126.com (C.W.); duxiliang@jlu.edu.cn (X.D.); jiluleilin@126.com (L.L.); gaowenwen@jlu.edu.cn (W.G.); liuguowen2008@163.com (G.L.); lixinwei100@126.com (X.L.)

**Keywords:** heat stress, *L*-Arginine, bovine neutrophil, apoptosis, nuclear factor erythroid 2-related factor 2

## Abstract

Heat stress severely impairs immune function and threatens dairy cow health and productivity. Although heat stress suppresses bovine neutrophil (PMN) phagocytosis, its effects governing PMN survival remain unclear. *L*-arginine (*L*-Arg) exerts immunomodulatory and cytoprotective effects, yet its role in repairing heat-damaged PMN has not been defined. In this study, an in vitro heat stress model (42 °C for 2.5 h) was established. Heat stress significantly reduced cell viability, induced abnormal nuclear morphology, and triggered apoptotic signaling, accompanied by severe oxidative stress (elevated reactive oxygen species (ROS)/Malondialdehyde (MDA), decreased SOD/glutathione peroxidase (GSH-Px)). The ROS scavenger N-Acetylcysteine (NAC) mitigated both oxidative stress and apoptosis, confirming oxidative stress as a core mediator of PMN apoptosis induced by heat stress. Pretreatment with 4 mmol/L *L*-Arg significantly alleviated heat-induced oxidative damage and apoptosis. Mechanistically, in bovine PMN, we first demonstrated that heat stress blocks nuclear factor erythroid 2-related factor 2 (NFE2L2) nuclear translocation; inhibition of NFE2L2 (ML385) abolished *L*-Arg’s protection, verifying the NFE2L2-dependent ROS scavenging pathway. In conclusion, this study reveals that heat stress induces bovine PMN oxidative injury and apoptosis by suppressing NFE2L2-mediated ROS scavenging, and *L*-Arg restores PMN viability and immune resilience by reactivating the NFE2L2 antioxidant pathway. These findings provide a targeted *L*-Arg supplement strategy to improve PMN survival and disease resistance in heat-stressed dairy cows.

## Simple Summary

Heat stress compromises dairy cow health and productivity by impairing innate immune function. Polymorphonuclear neutrophils (PMNs) serve as the first line of immune defense, and their functional integrity is critical for resisting pathogenic challenge during thermal stress. This study demonstrates that *L*-arginine (*L*-Arg) enhances the resilience of bovine PMN against heat stress by activating the nuclear factor erythroid 2-related factor 2 (NFE2L2) signaling pathway, which strengthens intracellular antioxidant capacity and reduces excessive reactive oxygen species (ROS) accumulation. These results identify *L*-Arg as an effective nutritional approach to mitigate heat stress-induced immune dysfunction in dairy cows, providing a practical strategy to support animal health and welfare under thermal challenge.

## 1. Introduction

With global warming, heat stress has become a critical constraint to the dairy industry, causing massive economic losses via reduced milk yield, reproductive disorders, and elevated infectious disease risk (e.g., mastitis) due to innate immune collapse [1,2,3]. Dairy cows are highly susceptible to heat stress owing to limited heat dissipation, leading to a pronounced imbalance between heat production and loss [3]. Heat stress not only reduces immune cell counts but also directly impairs PMN survival and antimicrobial function, which is the root cause of immunosuppression in summer [1,4].

PMNs are the most abundant leukocytes and the first line of innate immune defense against invading pathogens [5]. Previous studies showed heat stress suppresses PMN phagocytosis and migration [6,7] and reduces PMN counts in heat-stressed cows in summer [4]. However, the molecular mechanism linking heat stress to PMN apoptosis and immune dysfunction remains largely unknown, hindering the development of targeted immune protection strategies for dairy cows with heat stress.

Oxidative stress is a key mediator of heat-induced cellular damage [8]. In bovine mammary and intestinal epithelial cells, heat stress triggers ROS overproduction and mitochondrial injury [9,10,11]. In immune cells, excessive ROS directly determines neutrophil lifespan and functional integrity [12]. However, whether ROS accumulation drives heat-induced PMN apoptosis in dairy cows, and the upstream regulatory pathway, remains unaddressed.

Nuclear factor erythroid 2-related factor 2 (NFE2L2) is the master regulator of cellular antioxidant defense. It translocates into the nucleus to activate antioxidant genes (*NQO1*, *GCLC*, *GCLM*) and antioxidant enzymes (superoxide dismutase (SOD), heme oxygenase-1 (HMOX1)) to maintain redox homeostasis [13]. Impaired NFE2L2 signaling directly reduces cellular heat tolerance; however, its role in bovine PMN under heat stress has not been reported.

*L*-Arginine (*L*-Arg), a conditionally essential amino acid, exhibits robust cytoprotective and antioxidant properties across diverse cell types. In bovine intestinal epithelial cells, *L*-Arg mitigates heat stress damage by activating NFE2L2 and enhancing glutathione synthesis [10]. In non-ruminants, *L*-Arg protects against hepatic oxidative stress via NFE2L2-dependent upregulation of antioxidant enzymes in rats [13,14]. Studies in human PMN have shown that *L*-Arg supplementation induces nitric oxide synthesis, stabilizes intracellular Ca^2+^ homeostasis, and suppresses oxidative stress-related cell death [15]. However, in bovine PMN, its role and molecular mechanism in heat-stressed PMN remain unexplored.

We hypothesized that heat stress induces bovine PMN apoptosis via oxidative stress by inhibiting NFE2L2 nuclear translocation, and *L*-Arg attenuates these impairments by reactivating the NFE2L2-ROS scavenging axis. This study aims to clarify the mechanism of heat-induced PMN immune injury and provide a scientific basis for nutritional interventions to sustain dairy cow innate immunity under heat stress. 

## 2. Materials and Methods

### 2.1. Animals and Primary Bovine PMN Isolation

All experimental procedures (No. SY202401007) were approved by the Animal Care and Use Ethics Committee of Jilin University (Changchun, China). Cows were managed humanely in accordance with the Guidelines for the Care and Use of Agricultural Animals in Research and Teaching, 3rd Edition [16]. Five healthy mid-lactation Holstein cows (parity 2–3, 120–150 days in milk, body weight 650–750 kg) were selected during winter, and all cows were free of clinical diseases (e.g., mastitis, fever, and metabolic disorders) and had no history of antibiotic use within 2 weeks prior to blood collection. The cows were housed in a standardized barn with consistent feeding and management conditions (ambient temperature 18–22 °C, relative humidity 50–60%, ad libitum access to clean water and total mixed ration) to ensure the stability of physiological status.

PMNs were isolated from the jugular vein of each selected cow. The experiments were conducted using independent biological replicates from individual animals. Briefly, 20–30 mL of jugular venous blood was collected aseptically using heparinized vacuum blood collection tubes (10 U/mL heparin). The blood samples were immediately transported to the laboratory at room temperature within 1 h after collection to avoid PMN activation or damage caused by prolonged storage or temperature fluctuation. PMNs were purified using a commercial isolation kit (P9400, Solarbio Science and Technology Co., Ltd., Beijing, China) following the manufacturer’s instructions. All isolation steps were performed at room temperature to minimize temperature-induced alterations in PMN physiology. The purity and viability of isolated PMNs were verified by Wright-Giemsa staining and trypan blue exclusion test, respectively. Only PMNs with purity ≥ 95% and viability ≥ 90% were used for subsequent in vitro heat stress experiments.

### 2.2. Cell Treatment

To determine the optimal heat stress duration, a control group was maintained at 37 °C, while the experimental groups were exposed to 42 °C in a 5% CO_2_ incubator for 0.5, 1.0, 1.5, 2.0, 2.5, or 3.0 h [9]. Compared with the control group, heat exposure at 42 °C for 2.5 h significantly reduced PMN viability. Therefore, 2.5 h was selected as the standard heat stress duration for all subsequent in vitro experiments.

Physiological plasma *L*-Arg concentrations in calves range from 148.5 to 219.5 μmol/L [17], and clinical concentrations in dairy cows can reach 2.9, 7.2, or even 12.5 mmol/L without obvious toxicity [18,19,20]. Based on the optimized heat stress duration, PMNs were pretreated with 2, 4, 6, 8, or 10 mmol/L *L*-Arg (cat. No. A0013; Solarbio Science and Technology Co., Ltd.) for 30 min prior to heat exposure to identify the optimal protective concentration. Compared with the heat stress group, 4 mmol/L *L*-Arg exerted the most pronounced cytoprotective effect on cell viability; therefore, 4 mmol/L was used as the working concentration for *L*-Arg treatment.

For ROS inhibition experiments, PMNs were pretreated with the antioxidant N-acetylcysteine (NAC; cat. No. S1623; Selleck, Houston, TX, USA) at 4 mmol/L for 2 h before heat stress [21]. For NFE2L2 inhibition assays, PMNs were pretreated with the specific NFE2L2 inhibitor ML385 (5 μmol/L; cat. No. S8790; Selleck) for 2 h [22].

### 2.3. Cell Viability Assay

PMN viability was assessed using an Enhanced Cell Counting Kit-8 (CCK-8, cat. No. ST1007; Saint-Bio Biotechnology Co., Ltd., Shanghai, China) according to the manufacturer’s protocol. Briefly, 1 × 10^5^ PMNs (100 μL per well) were seeded in 96-well plates. After treatment, 10 μL of enhanced CCK-8 solution was added to each well. Absorbance at 450 nm was measured using a Multiskan FC microplate reader (cat. No. 51119180ET; Thermo Fisher Scientific, Waltham, Massachusetts, USA). Cell viability was calculated as follows: cell viability (%) = (mean absorbance of treatment group/mean absorbance of control group) × 100%.

### 2.4. Protein Extraction and Western Blotting

Nuclear and cytoplasmic proteins were extracted using commercial kits (cat. No. P0027; Beyotime, Shanghai, China) following the manufacturer’s instructions. Total protein was extracted using a total protein extraction kit (Sangon Biotech, Shanghai, China). Protein concentrations were quantified using the bicinchoninic acid (BCA) assay (cat. No. C510003; Sangon Biotech; cat. No. P1511; Applygen, Beijing, China). Equal amounts of protein (30 μg per sample) were separated by 15% SDS-PAGE and then electrotransferred onto polyvinylidene difluoride (PVDF) membranes. Membranes were blocked with 5% BSA in Tris-buffered saline containing 0.1% Tween-20 (TBST) for 4 h, then incubated overnight at 4 °C with primary antibodies against HSP70 (1:1000; bs-0244R; Bioss Antibodies, Beijing, China), cleaved-caspase 3 (1:1000; A19654; ABclonal, Wuhan, China), caspase 3 (1:1000; A19654; ABclonal), BAX (1:1000; ab32503; Abcam, Cambridge, UK), BCL2 (1:1000; bsm-33047M; Bioss Antibodies), NFE2L2 (1:1000; AF0639; Affinity, Changzhou, China), HMOX1 (1:1000; 10701-1-AP; Proteintech, Rosemont, IL, USA), LMNB1 (1:1000; AF5161; Affinity), and β-actin (1:2000; ab8226; Abcam). Membranes were then washed three times with TBST and incubated with horseradish peroxidase-conjugated anti-rabbit (1:5000; SA00001-2; Proteintech) or anti-mouse (1:5000; SA00001-1; Proteintech) secondary antibodies for 45 min at room temperature. After three additional TBST washes, immunoreactive bands were visualized using enhanced chemiluminescence (ECL) reagent (WBKLS0500; Millipore, Burlington, MA, USA). β-actin served as the loading control. All antibodies cross-reacted with bovine target proteins. Band images were captured using a Protein Simple Imager (ProteinSimple, San Jose, CA, USA), and densitometric quantification was performed using Image-Pro Plus 6.0 software (Media Cybernetics, Rockville, MD, USA).

### 2.5. Neutrophil Apoptosis Assay

Apoptotic rates were quantified using an Annexin V-FITC/propidium iodide (PI) apoptosis detection kit (cat. No. 556547; BD Biosciences, San Jose, CA, USA). PMNs were washed twice with cold PBS, resuspended in 100 μL of 1× binding buffer, then incubated with 5 μL Annexin V-FITC and 5 μL PI for 15 min in the dark. After incubation, 400 μL of 1× binding buffer was added to each tube. Apoptosis was analyzed via flow cytometry BD FACSCalibur (Becton Dickinson, Franklin Lakes, NJ, USA), and data were processed using the corresponding software. Total apoptotic cells were defined as Annexin V-positive cells; early and late apoptotic cells were quantified from the lower-right and upper-right quadrants, respectively.

### 2.6. Immunofluorescence Assay

PMNs (1 × 10^6^/mL) were seeded onto laser confocal dishes (cat. No. 801002; NEST Biotechnology, Wuxi, China) precoated with 0.1 mg/mL poly-d-lysine (cat. No. P1149; Sigma-Aldrich, Burlington, MA, USA). After treatment, cells were washed twice with PBS, fixed with 4% paraformaldehyde for 20 min, then washed once with cold PBS. Nuclei were stained with Hoechst 33258 (cat. No. C1017; Beyotime, Shanghai, China) for 20 min. Samples were sealed with glycerol and imaged using a laser confocal microscope (FV500; Olympus, Tokyo, Japan). Apoptotic nuclei were identified using condensed or fragmented hyperfluorescent staining.

### 2.7. Intracellular ROS Measurement

Intracellular ROS levels were detected using a reactive oxygen species assay kit (cat. No. S0033S; Beyotime). After treatment, PMNs were incubated with dichlorofluorescin diacetate (DCFH-DA) for 30 min. Cells were collected via centrifugation, washed twice with cold PBS, resuspended in 200 μL PBS, and analyzed using flow cytometry (Becton Dickinson). Fluorescence intensity was proportional to intracellular ROS levels.

### 2.8. Quantitative Reverse-Transcription PCR (qRT-PCR)

Total RNA was extracted from PMN using TRIzol reagent (cat. No. 15596026; Invitrogen, Waltham, MA, USA) following the manufacturer’s instructions. RNA concentration and integrity were verified using a K5500 microspectrophotometer (Beijing Kaiao, Beijing, China) and 1% agarose gel electrophoresis. One microgram of total RNA was reverse-transcribed into cDNA using a PrimeScript Reverse Transcriptase Kit (cat. No. MR05101; Monad, Suzhou, China). Gene-specific primers were designed using Primer Express 3.0 software (Applied Biosystems, Waltham, MA, USA) and are listed in Table 1. qRT-PCR was performed using SYBR Green Plus reagent (cat. No. 4913850001; Roche, Basel, Switzerland) on a 7500 Real-Time PCR System (Applied Biosystems). The thermal cycling conditions were 95 °C for 3 min, followed by 40 cycles of 95 °C for 15 s and 60 °C for 1 min. Relative mRNA expression was calculated using the 2^−ΔΔCT^ method (De Ketelaere et al., 2006) and normalized to the geometric mean of ACTB and YWHAZ. Each treatment was performed in triplicate.

### 2.9. Antioxidant Index Determination

The activities of superoxide dismutase (SOD), glutathione peroxidase (GSH-Px), and Malondialdehyde (MDA) content were measured using commercial assay kits (cat. Nos. S0101S, S0057S, S0131S; Beyotime) according to the manufacturer’s protocols. Results were normalized to total protein concentration.

### 2.10. Statistical Analysis

Data normality and variance homogeneity were evaluated using the Shapiro–Wilk test and Levene’s test, respectively. Outliers were identified using the interquartile range method; no outliers were excluded from the analysis. Two-way ANOVA followed by Bonferroni post hoc testing was used to analyze the interactive effects of two treatments (HS vs. NAC; HS vs. *L*-Arg; *L*-Arg vs. ML385) on apoptotic rate, antioxidant enzyme activities, and gene/protein expression. One-way ANOVA with Bonferroni correction or independent-samples *t*-test was used for single-factor comparisons. This study included 5 biological replicates, with 3 technical replicates for each sample. The values shown in the figure represent the average of technical replicates for each biological sample. All statistical analyses were performed using GraphPad Prism 8.3.0 software. Data are presented as means ± standard error of the mean (SEM). *p* < 0.05 indicates statistical significance, and *p* < 0.01 indicates high statistical significance. Different lowercase letters denote significant differences between groups (*p* < 0.05).

## 3. Results

### 3.1. Heat Stress Promotes Apoptosis in Bovine PMNs In Vitro

**Figure 1:**
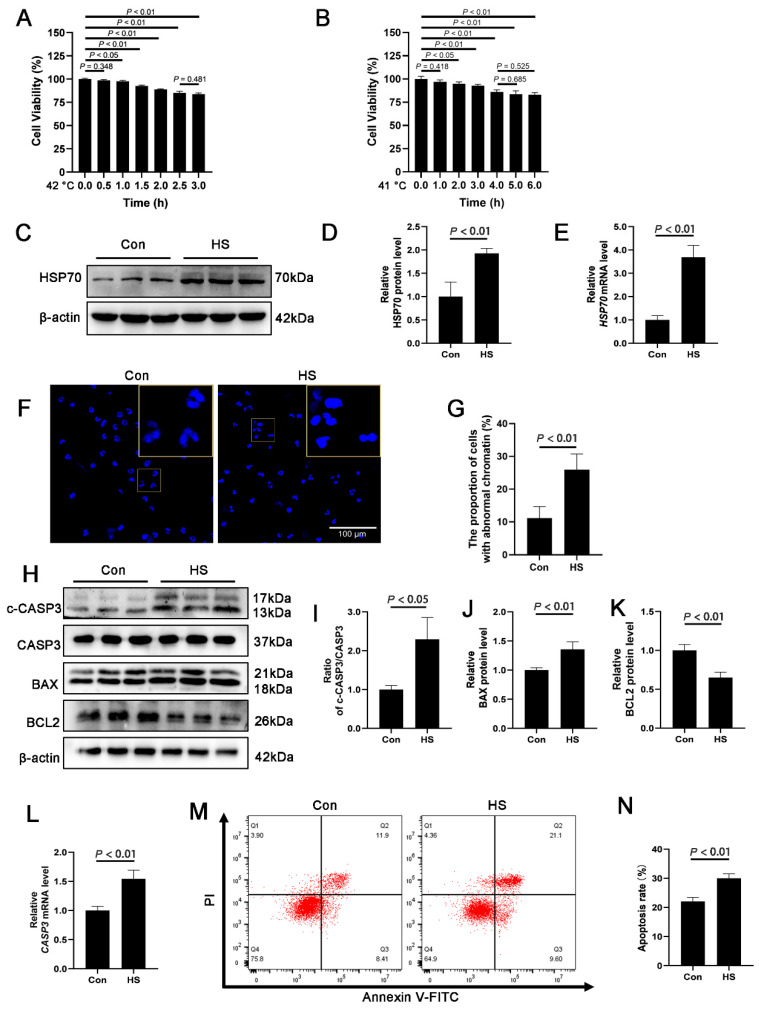
Effects of heat stress on bovine PMN apoptosis in vitro (n = 5). (**A**) The effect of different times of heat treatment (42 °C) on the cell viability. (**B**) The effect of different times of heat treatment (41 °C) on the cell viability. (**C**,**D**) Protein abundance of HSP70 (the original western blot pictures can be found in File S1). (**E**) mRNA abundance of *HSP70*. (**F**,**G**) Immunofluorescence display of nuclear changes in PMN cells under heat stress. Magnification = 400×. Scale bar = 100 μm. Nuclei/Hoechst 33258 is in blue. (**H**–**K**) Protein abundance of c-CASP3, CASP3, BAX, and BCL2 (the original western blot pictures can be found in File S1). (**L**) mRNA abundance of *CASP3*. (**M**,**N**) Flow cytometry analysis of PMN apoptosis after heat stress. Data were analyzed with a one-way ANOVA in (**A**,**B**) or independent-samples *t*-tests in (**D**–**N**). All data are expressed as means ± SEM; *p* < 0.05, indicating a significant difference; *p* < 0.01, indicating an extremely significant difference.

Compared with the control group, heat exposure at 42 °C for 2.5 h significantly reduced PMN viability (Figure 1A, *p* < 0.01). No significant difference was observed between the 2.5 h and 3.0 h heat-treated groups (*p* = 0.48), validating 2.5 h as the optimal heat stress duration. Treatment of PMNs at a relatively mild temperature of 41 °C for 4 h also significantly suppressed their viability (Figure 1B, *p* < 0.01), indicating that acute heat shock produces identical outcomes to chronic environmental heat stress. Heat stress markedly upregulated HSP70 protein and mRNA expression (Figure 1C–E; *p* < 0.01), confirming successful establishment of the in vitro heat stress model.

Nuclear staining showed prominent chromatin condensation and fragmentation in heat-stressed PMNs, which is indicative of increased apoptosis (Figure 1F,G). Furthermore, heat stress significantly enhanced the protein abundance of cleaved-caspase 3 (c-CASP3) and the c-CASP3/CASP3 ratio (Figure 1H,I; *p* = 0.02), as well as the mRNA levels of *CASP3* (Figure 1L; *p* < 0.01), suggesting the activation of caspase-dependent apoptotic signaling. Consistently, the pro-apoptotic BAX protein was upregulated (Figure 1H,J; *p* < 0.01), whereas the anti-apoptotic BCL2 protein was downregulated (Figure 1H,K; *p* < 0.01). Finally, flow cytometric analysis further confirmed that heat stress significantly increased the total apoptotic rate of PMNs (Figure 1M,N; *p* < 0.01).

### 3.2. Oxidative Stress Mediates Heat Stress-Induced Bovine PMN Apoptosis

**Figure 2:**
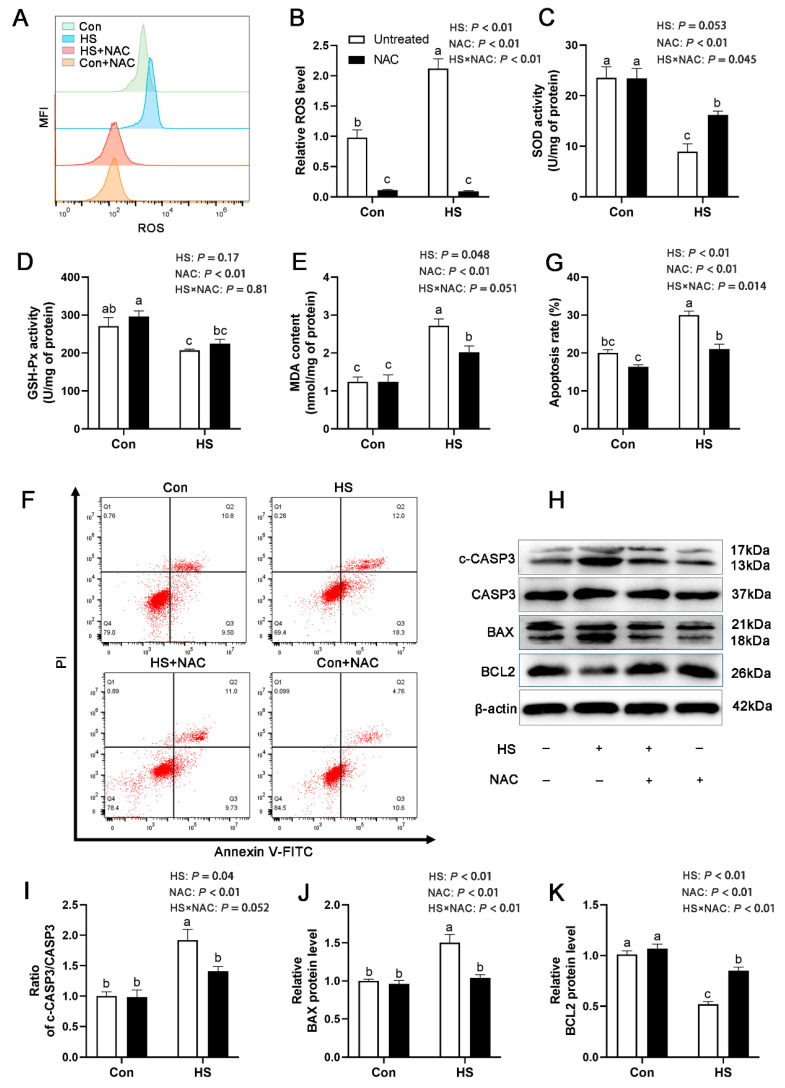
Effects of heat stress on the oxidative stress state of bovine PMN in vitro (n = 5). (**A**,**B**) Flow cytometry analysis of intracellular ROS. (**C**–**E**) The cellular activity of SOD, GSH-Px, and the content of MDA. (**F**,**G**) Flow cytometry analysis of PMN apoptosis after different treatments. (**H**–**K**) Protein abundances of c-CASP3, CASP3, BAX, and BCL2. Data were analyzed with two-way ANOVA. All data are expressed as means ± SEM. Bars with different lowercase letters (a–c) indicate a significant difference (*p* < 0.05) (the original western blot pictures can be found in File S1).

Heat stress significantly increased intracellular ROS accumulation (Figure 2A,B; *p* < 0.01), reduced SOD and GSH-Px activities (Figure 2C,D; *p* < 0.01), and elevated MDA content (Figure 2E; *p* < 0.01). The ROS scavenger NAC reversed these effects, restoring SOD activity (*p* < 0.01) and reducing MDA levels (*p* = 0.049), although it did not significantly affect GSH-Px activity (*p* = 0.377). NAC also attenuated heat stress-induced apoptosis, as evidenced by reduced apoptotic rate (Figure 2F,G; *p* < 0.01), decreased c-CASP3 and BAX expression, and increased BCL2 expression (Figure 2H–K; *p* < 0.01). These results demonstrate that oxidative stress plays a central role in mediating heat stress-induced PMN apoptosis.

### 3.3. L-Arg Inhibits Heat Stress-Induced Apoptosis in Bovine PMNs

**Figure 3:**
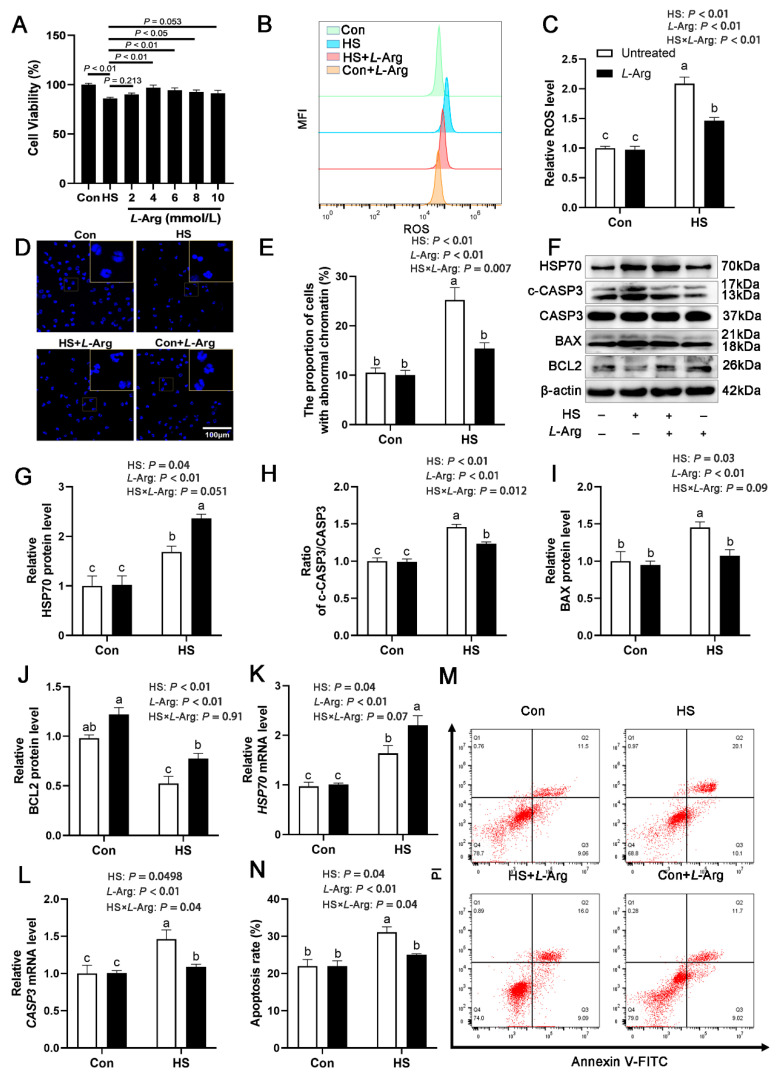
Effects of *L*-Arg on bovine PMN induced by heat stress viability and nuclear morphology in vitro (n = 5). (**A**) The effect of different concentrations of *L*-Arg on cell viability. (**B**,**C**) Flow cytometry analysis of intracellular ROS. (**D**,**E**) Immunofluorescence experiments showed that *L*-Arg improved nuclear morphology of PMN induced by heat stress. Magnification = 400×. Scale bar = 100 μm. Nuclei/Hoechst 33258 is in blue. (**F**–**J**) Protein abundances of HSP70, c-CASP3, CASP3, BAX, and BCL2 (the original western blot pictures can be found in File S1). (**K**,**L**) mRNA abundance of *HSP70* and *CASP3*. (**M**,**N**) Flow cytometry analysis of PMN apoptosis after different treatments. Data were analyzed with one-way ANOVA in (**A**) or two-way ANOVA in (**C**–**N**). All data are expressed as means ± SEM. *p* < 0.05, indicating a significant difference; *p* < 0.01, indicating an extremely significant difference. Bars with different lowercase letters (a–c) indicate a significant difference (*p* < 0.05).

CCK-8 assay showed that 4–8 mmol/L *L*-Arg pretreatment significantly improved the viability of heat-stressed PMNs, with maximal protection at 4 mmol/L (Figure 3A; *p* < 0.01). Thus, 4 mmol/L *L*-Arg was used for subsequent experiments. *L*-Arg pretreatment markedly reduced intracellular ROS levels (Figure 3B,C; *p* < 0.01) and alleviated heat stress-induced nuclear morphological damage (Figure 3D,E). *L*-Arg also upregulated HSP70 protein and mRNA expression (Figure 3F,G,K; *p* = 0.04 or *p* < 0.01), enhanced BCL2 abundance (Figure 3F,J; *p* < 0.01), and suppressed c-CASP3 and BAX expression (Figure 3F,H,I; *p* < 0.01 or *p* = 0.049). Correspondingly, *L*-Arg significantly reduced the total apoptotic rate of heat-stressed PMNs (Figure 3M,N; *p* = 0.03). These results demonstrate that *L*-Arg can alleviate heat stress-induced PMN apoptosis by reducing oxidative stress.

### 3.4. Heat Stress Impairs the Nuclear Translocation of NFE2L2 in Bovine Neutrophils

**Figure 4:**
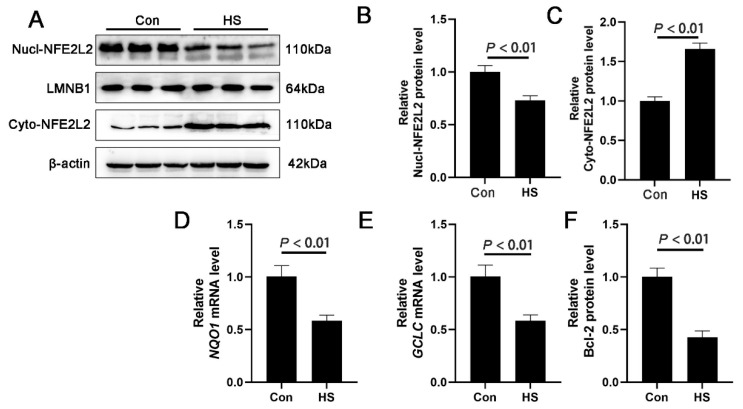
Impairment of NFE2L2 nuclear translocation of bovine PMN induced by heat stress in vitro (n = 5). (**A**–**C**) Protein abundances of Nucl-NFE2L2 and Cyto-NFE2L2 in Con and HS group (the original western blot pictures can be found in File S1). (**D**–**F**) mRNA abundance of *NQO1*, *GCLC* and *GCLM*. Data were analyzed with an independent-samples *t*-test in (**B**–**F**). All data are expressed as means ± SEM. *p* < 0.05, indicating a significant difference; *p* < 0.01, indicating an extremely significant difference.

Heat stress significantly decreased nuclear NFE2L2 (Nucl-NFE2L2) and increased cytoplasmic NFE2L2 (Cyto-NFE2L2) protein levels (Figure 4A–C; *p* < 0.01), indicating impaired nuclear translocation of NFE2L2. Consistently, the mRNA abundances of NQO1, GCLC and GCLM significantly decreased after heat stress (Figure 4D–F; *p* < 0.01).

### 3.5. L-Arg Alleviates Heat Stress-Induced Oxidative Stress by Activating the NFE2L2 Signaling Pathway

**Figure 5:**
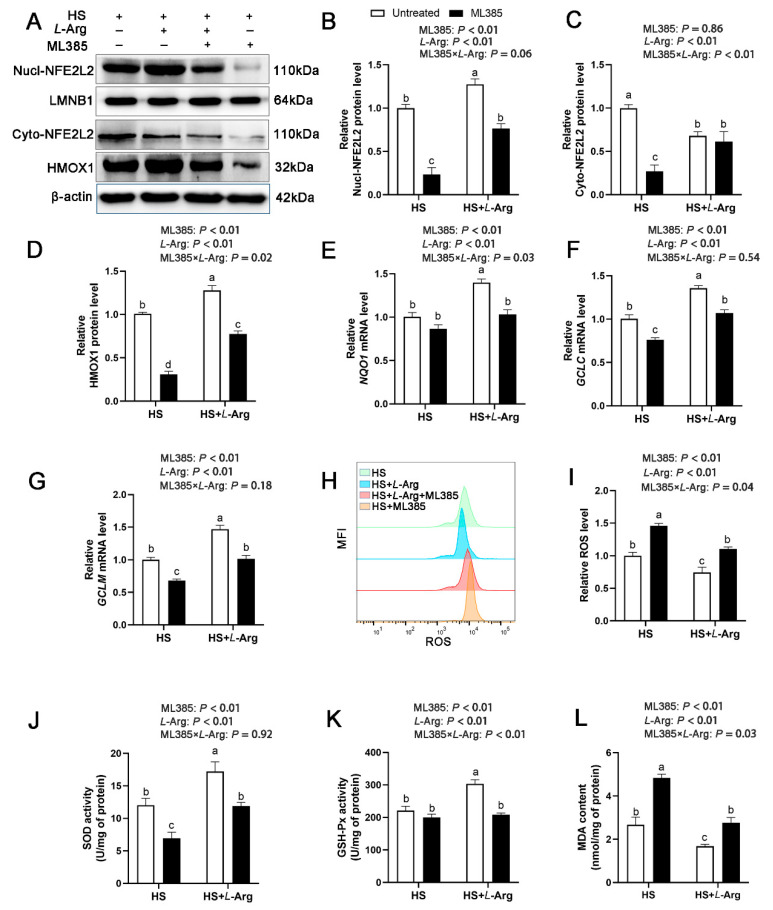
Role of NFE2L2 signaling pathway in *L*-Arg regulation of bovine PMN oxidative stress induced by heat stress in vitro (n = 5). (**A**–**D**) Protein abundances of Nucl-NFE2L2, Cyto-NFE2L2 and HMOX1 in HS, HS + *L*-Arg, HS + *L*-Arg + ML385, and HS + ML385 (the original western blot pictures can be found in File S1). (**E**–**G**) mRNA abundance of *NQO1*, *GCLC* and *GCLM*. (**H**,**I**) Flow cytometry analysis of intracellular ROS. (**J**–**L**) The cellular activities of SOD, GSH-Px and the content of MDA. Data were analyzed with two-way ANOVA in (**B**–**L**). All data are expressed as means ± SEM. Bars with different lowercase letters (a–d) indicate a significant difference (*p* < 0.05).

*L*-Arg treatment markedly promoted NFE2L2 nuclear translocation in heat-stressed PMNs (Figure 5A–C; *p* < 0.01), upregulated HMOX1 expression (Figure 5A,D; *p* < 0.01) and elevated the abundance of downstream antioxidant genes *NQO1*, *GCLC* and *GCLM* (Figure 5E–G; *p* < 0.01). *L*-Arg also reduced ROS and MDA accumulation (Figure 5H,I,L; *p* < 0.01) and enhanced SOD and GSH-Px activities (Figure 5J,K; *p* < 0.01). However, the NFE2L2 inhibitor ML385 abolished *L*-Arg-induced NFE2L2 nuclear translocation, HMOX1 upregulation (Figure 5A–D; *p* < 0.01) and the elevated mRNA levels of *NQO1*, *GCLC* and *GCLM* (Figure 5E–G; *p* < 0.01). Moreover, ML385 blocked *L*-Arg-mediated antioxidant responses, exacerbating oxidative stress (Figure 5H–L; *p* < 0.01). These results confirm that the antioxidant effects of *L*-Arg in heat-stressed PMNs are strictly NFE2L2-dependent.

### 3.6. NFE2L2 Signaling Mediates the Protective Effect of L-Arg Against Heat Stress-Induced Apoptosis in Bovine PMNs

**Figure 6:**
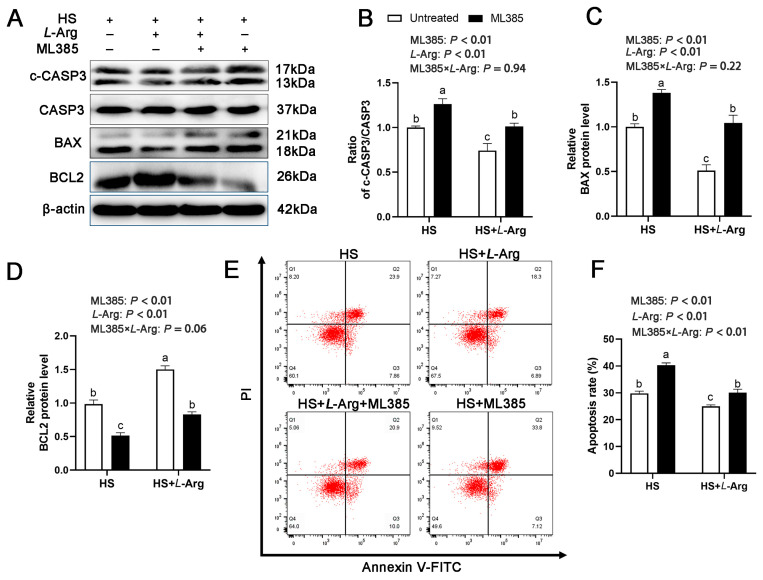
Role of NFE2L2 signaling pathway in *L*-Arg regulation of bovine PMN apoptosis induced by heat stress in vitro (n = 5). (**A**–**D**) Protein abundances of c-CASP3, CASP3, BAX, and BCL2 in HS, HS + *L*-Arg, HS + *L*-Arg + ML385, and HS + ML385 (the original western blot pictures can be found in File S1). (**E**,**F**) Flow cytometry analysis of PMN apoptosis after different treatments. Data were analyzed with two-way ANOVA in (**B**–**D**,**F**). All data are expressed as means ± SEM. Bars with different lowercase letters (a–c) indicate a significant difference (*p* < 0.05).

*L*-Arg treatment markedly decreased the c-CASP3/CASP3 ratio, BAX abundance, and apoptotic rate, while increasing BCL2 expression (Figure 6A–F; *p* < 0.01). The NFE2L2 inhibitor ML385 reversed the cytoprotective effects of *L*-Arg, further elevating the c-CASP3/CASP3 ratio, BAX expression, and apoptotic rate, while suppressing BCL2 abundance (Figure 6A–F; *p* < 0.01). These results indicate that the anti-apoptotic effect of *L*-Arg in heat-stressed PMNs is achieved by activating the NFE2L2 pathway to alleviate oxidative stress.

## 4. Discussion

This study demonstrates that heat stress induces oxidative damage and reduces viability in bovine PMNs by disrupting redox balance and that *L*-Arg counteracts these impairments by activating NFE2L2 signaling to reinforce antioxidant capacity and suppress ROS-mediated cellular injury. This study provides direct evidence that the NFE2L2-ROS axis serves as a core regulatory switch governing heat stress-induced apoptosis in bovine PMNs. These results provide a mechanistic explanation for the well-documented in vivo observation of reduced PMN counts and weakened antimicrobial defense in heat-stressed dairy cows during summer [4,23] and establish a direct causal link between heat stress, PMN loss, and immunosuppression.

Heat stress is a well-known environmental trigger of ROS generation [8]. Excess ROS activates death receptor and endoplasmic reticulum injury pathways [24]. In murine PMNs, ROS directly triggers cell death signaling via ceramide generation and death receptor clustering [12]. In human umbilical vein endothelial cells, heat stress-induced ROS accumulation activates p53- and Ca^2+^-MPTP-dependent mitochondrial injury signaling [25]. Our results confirm that heat stress markedly increases ROS levels in bovine PMNs, reduces SOD and GSH-Px activities, impairs NFE2L2 nuclear translocation, and downregulates HMOX1 expression, ultimately leading to oxidative stress. These findings are consistent with previous reports, showing ROS accumulation in heat-stressed bovine mammary epithelial cells [11,26], hepatocytes [27], and camel PMN [28], but extend this paradigm to the key innate immune effector cells that determine mastitis resistance and overall immune resilience.

Our study shows that heat stress induces nuclear morphological abnormalities and reduces cell viability in bovine PMNs, accompanied by altered expression of cell fate regulatory proteins. These results align with in vivo observations of reduced PMN counts in summer heat-stressed cows [4] and impaired viability in heat-stressed bovine mononuclear cells, human, and dromedary camel immune cells [28,29,30,31]. Thus, oxidative stress is a key mediator of heat stress-induced PMN dysfunction, which may explain the immunosuppression and heightened disease susceptibility observed in heat-stressed dairy cows [23]. Since intact PMN survival is a prerequisite for normal immune effector performance, elevated PMN apoptosis under heat stress indirectly reflects impaired innate immune resilience, which provides partial evidence for compromised host defense. In addition, previous studies have demonstrated that heat stress impairs the phagocytic [6] and bactericidal capacities [32] of bovine PMNs and suppresses their chemotactic function [33] by altering circulating corticosterone levels. This may partially account for the weakened immune performance of heat-stressed dairy cows.

*L*-Arg is a conditionally essential amino acid widely used in dairy nutrition owing to its cost-effectiveness and multifaceted benefits [34]. It improves milk production and quality and enhances heat stress tolerance by boosting antioxidant defenses [35]. Our results show that *L*-Arg elevates SOD and GSH-Px activities, reduces ROS and MDA levels, and clears excessive free radicals in heat-stressed PMNs. This is the first report demonstrating that *L*-Arg can rescue bovine PMNs from heat-induced oxidative damage, expanding the application of *L*-Arg from epithelial protection to innate immune enhancement. This is consistent with *L*-Arg-mediated antioxidant effects in bovine intestinal epithelial cells [10] and other species [13,36]. *L*-Arg also modulates key regulatory proteins associated with cell survival, consistent with its cytoprotective role in ovine intestinal epithelial cells and human PMNs [15,37].

The antioxidant effects of *L*-Arg are tightly linked to NFE2L2 signaling. *L*-Arg suppresses Keap1 and Cul3 expression, promoting NFE2L2 nuclear translocation and subsequent upregulation of ARE-driven antioxidant genes, including GSH-Px, SOD, and HMOX1 [13]. In mice, *L*-Arg activates NFE2L2 to alleviate LPS-induced oxidative stress and cellular damage in C2C12 myotubes [38]. Our study provides definitive evidence in bovine PMN that *L*-Arg exerts its anti-stress and anti-apoptotic effects exclusively through NFE2L2-dependent signaling. Heat stress directly blocks NFE2L2 nuclear translocation, whereas *L*-Arg effectively restores this process; inhibition of NFE2L2 using ML385 completely abolishes all protective effects of *L*-Arg. These findings identify NFE2L2 as a necessary molecular target for *L*-Arg in alleviating heat stress in bovine innate immune cells, a novel mechanistic discovery that distinguishes the present work from previous studies focused only on epithelial cells.

The present study also has several limitations. The 42 °C, 2.5 h in vitro acute heat shock model cannot fully recapitulate the chronic, multi-factorial heat stress dairy cows undergo under natural farm conditions. A previous seasonal field trial indicated that dairy cows suffer a significant reduction in peripheral PMN quantity in hot summer seasons [4]. Notably, our data demonstrate that prolonged mild heat exposure at 41 °C also impairs PMN viability, demonstrating consistent cellular damage outcomes between acute thermal shock and sustained mild hyperthermia. While in vitro acute models simplify complex in vivo systemic factors, they serve as a standardized, controllable platform to dissect the direct intracellular molecular responses of PMNs to heat. Our findings lay a foundation for exploring the molecular mechanisms underlying heat stress in bovine PMNs, and further in vivo trials are required to validate these outcomes under chronic environmental heat stress. The 4 mmol/L *L*-Arg adopted in this study mitigates heat stress-triggered apoptosis of bovine PMNs under in vitro culture conditions. However, free *L*-Arg is degraded in the rumen, and only rumen-protected *L*-Arg can be absorbed by the body. Accordingly, this concentration cannot be directly adopted as the standard dosage for oral feeding or injection, yet it can serve as a reference index for *L*-Arg concentration measurement in in vivo trials. Further in vivo experiments are required to explore the exact administration concentration.

In summary, this study demonstrates that heat stress induces oxidative stress and subsequent apoptosis in bovine PMN by suppressing NFE2L2 nuclear translocation and impairing intracellular ROS-scavenging capacity. *L*-Arg protects bovine PMNs against heat stress-induced injury by restoring NFE2L2 nuclear translocation and activating NFE2L2-mediated antioxidant signaling, which enhances the expression of antioxidant genes *NQO1*, *GCLC*, and *GCLM*, improves SOD and GSH-Px activities, clears excessive ROS, reduces lipid peroxidation, and ultimately inhibits apoptotic signaling. These findings elucidate a novel molecular mechanism underlying heat stress-induced innate immune dysfunction in dairy cows and identify the NFE2L2-ROS-apoptosis axis as a key regulatory target. This work also establishes *L*-Arg as a promising nutritional strategy to improve neutrophil survival, immune resilience, and disease resistance in heat-stressed dairy cows, providing a mechanistic basis for nutritional intervention to alleviate heat stress damage in the dairy industry.

## Figures and Tables

**Table 1:** The primer sequences used for quantitative real-time PCR.

Gene	RefSeq	Primer Sequences (5′–3′)	Product Length (bp)
*HSP70*	XM_005911049.2	For: CCTTCCCTGGATTGCTCATGT	89
		Rev: TGATGGCTGATGAAAGGCAGA	
*CASP3*	NM_001077840.1	For: AGACAGACAGTGGTGCTGAG	101
		Rev: CCAGGAAAAGTAACCAGGTGCT	
*NQO1*	NM_001034535	For: CAACAGACCAGCCAATCA	144
		Rev: ACCTCCCATCCTTTCCTC	
*GCLC*	NM_001083674	For: CACAAATTGGCAGACAATGC	211
		Rev: GGCGACCTTCATGTTCTCAT	
*GCLM*	NM_001038143	For: TGGAGCAGCTGTATCAGTGG	198
		Rev: GAATGTCAGGGATGCTCTCC	
*ACTB*	NM_173979.3	For: CCTGCGGCATTCACGAAACTAC	273
		Rev: ACTCCTGCTTGCTGATCCACATC	
*YWHAZ*	NM_174814.2	For: CACCTACTCCGGACACAGAAC	200
		Rev: TGACCTACGGGCTCCTACAA	

## Data Availability

The original contributions presented in this study are included in the article. Further inquiries can be directed to the corresponding author.

## Supplementary Materials

The following supporting information can be downloaded at: https://www.mdpi.com/article/10.3390/vetsci13070628/s1, File S1: Original images of Western-blotting.

## Abbreviations

HS = Heat stress; *L*-Arg = *L*-Arginine; PMN = Polymorphonuclear neutrophil; c-CASP3 = Cleaved-caspase 3; NFE2L2 = Nuclear factor erythroid 2-related factor 2; ROS = Reactive oxygen species; SOD = Superoxide Dismutase; GSH-Px = Glutathione Peroxidase; MDA = Malondialdehyde; NAC = N-Acetylcysteine; HMOX1 = Heme oxygenase-1; MFI = Mean fluorescence intensity; BCL2 = B-cell lymphoma 2; BAX = BCL2-associated X protein; NQO1 = NAD(P)H dehydrogenase (quinone) 1; GCLC = Glutamate-cysteine ligase catalytic subunit; GCLM = Glutamate-cysteine ligase modifier subunit.
