# Supplementary figures and images for: L-Arginine Attenuates Heat Stress-Induced Oxidative Damage and Apoptosis in Bovine Neutrophils via NFE2L2-Mediated ROS Scavenging
Source: Vet Sci. 2026 Jun 27;13(7):628. doi: 10.3390/vetsci13070628 (PMC13417081; doi:10.3390/vetsci13070628)

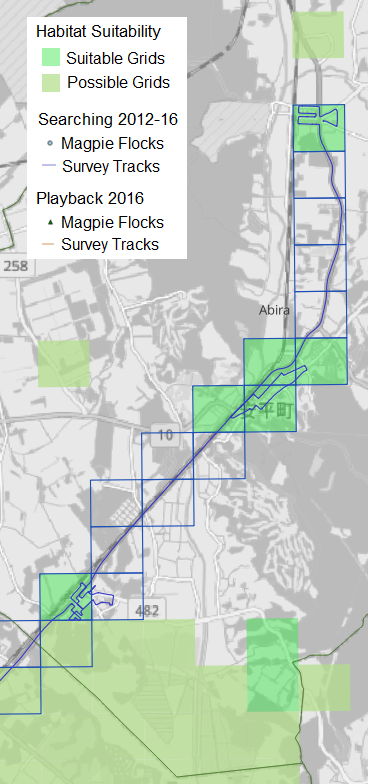

Supplement: Supplementary file 1 [file vetsci-13-00628-s001.zip › D1_Abiora.png]

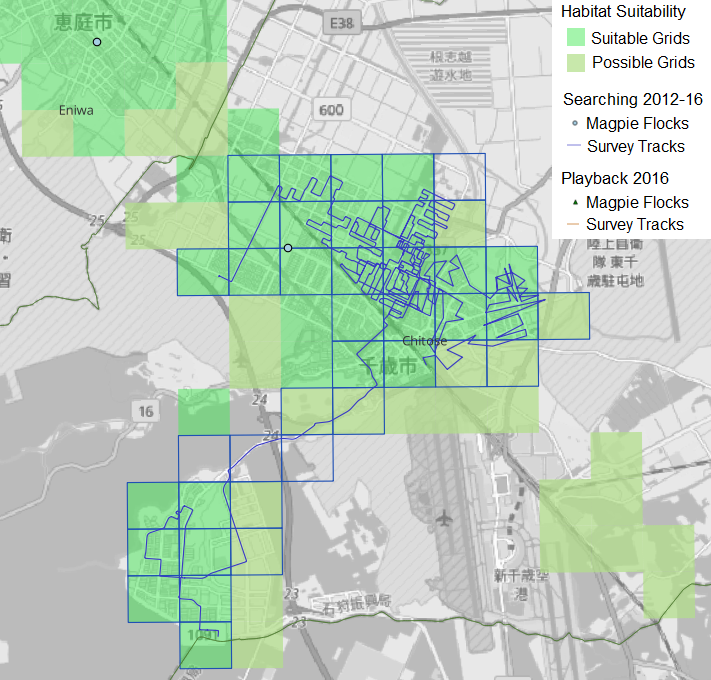

Supplement: Supplementary file 1 [file vetsci-13-00628-s001.zip › D2_Chitose.png]

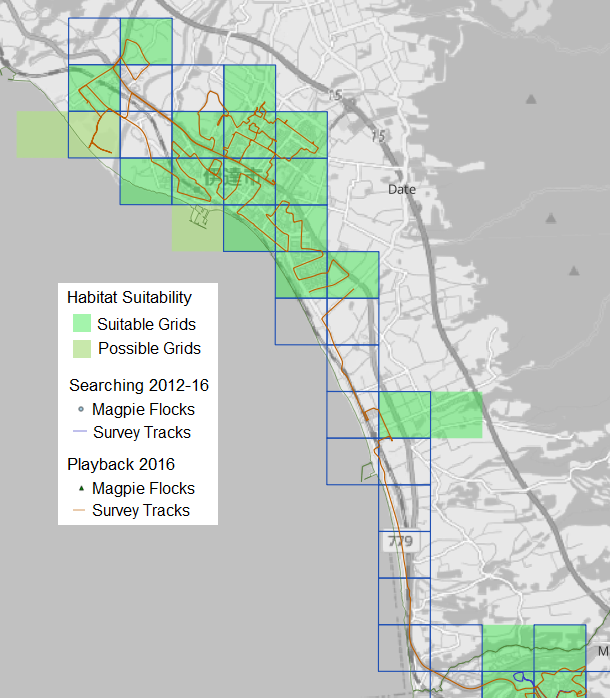

Supplement: Supplementary file 1 [file vetsci-13-00628-s001.zip › D3_Date.png]

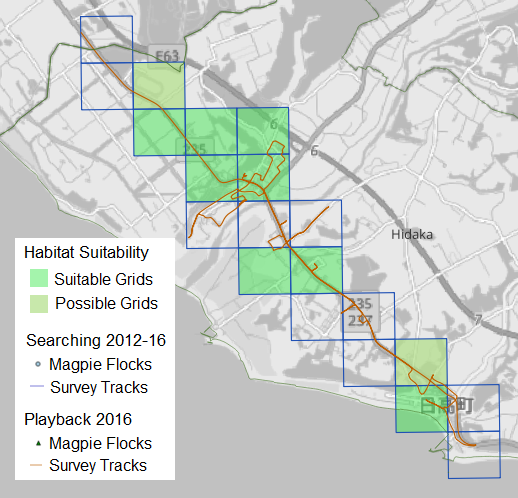

Supplement: Supplementary file 1 [file vetsci-13-00628-s001.zip › D4_Hidaka.png]

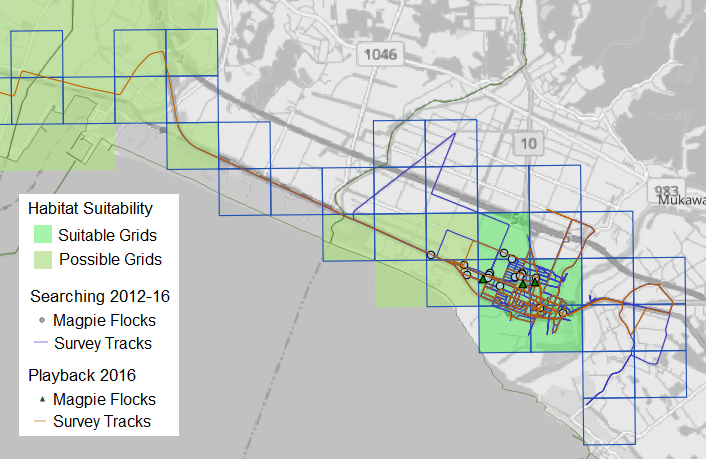

Supplement: Supplementary file 1 [file vetsci-13-00628-s001.zip › D5_Mukawa.png]

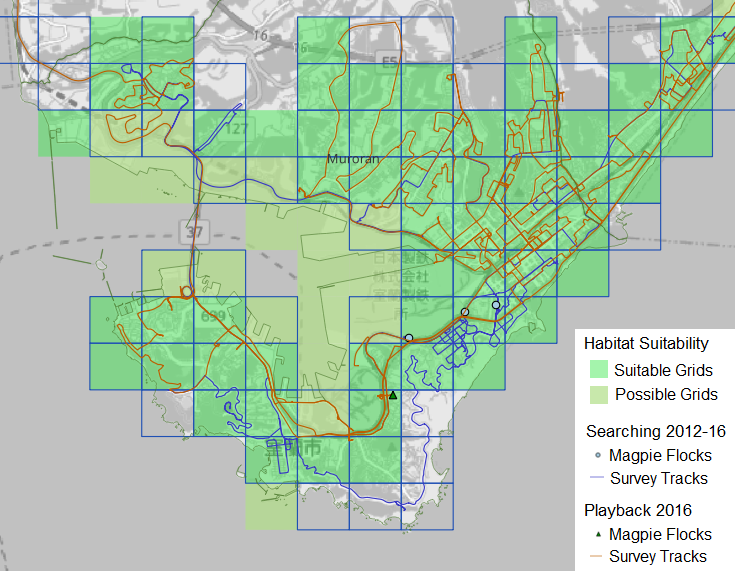

Supplement: Supplementary file 1 [file vetsci-13-00628-s001.zip › D6_Muroran.png]

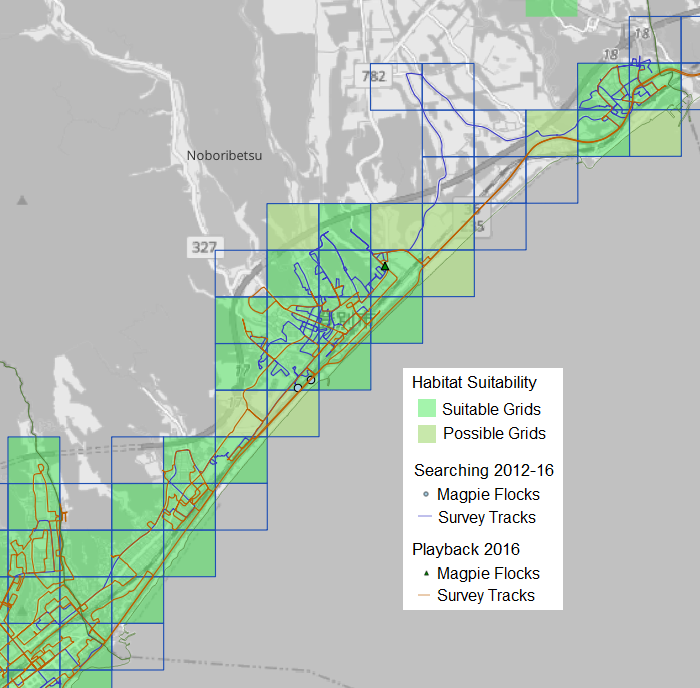

Supplement: Supplementary file 1 [file vetsci-13-00628-s001.zip › D7_Noboribetsu.png]

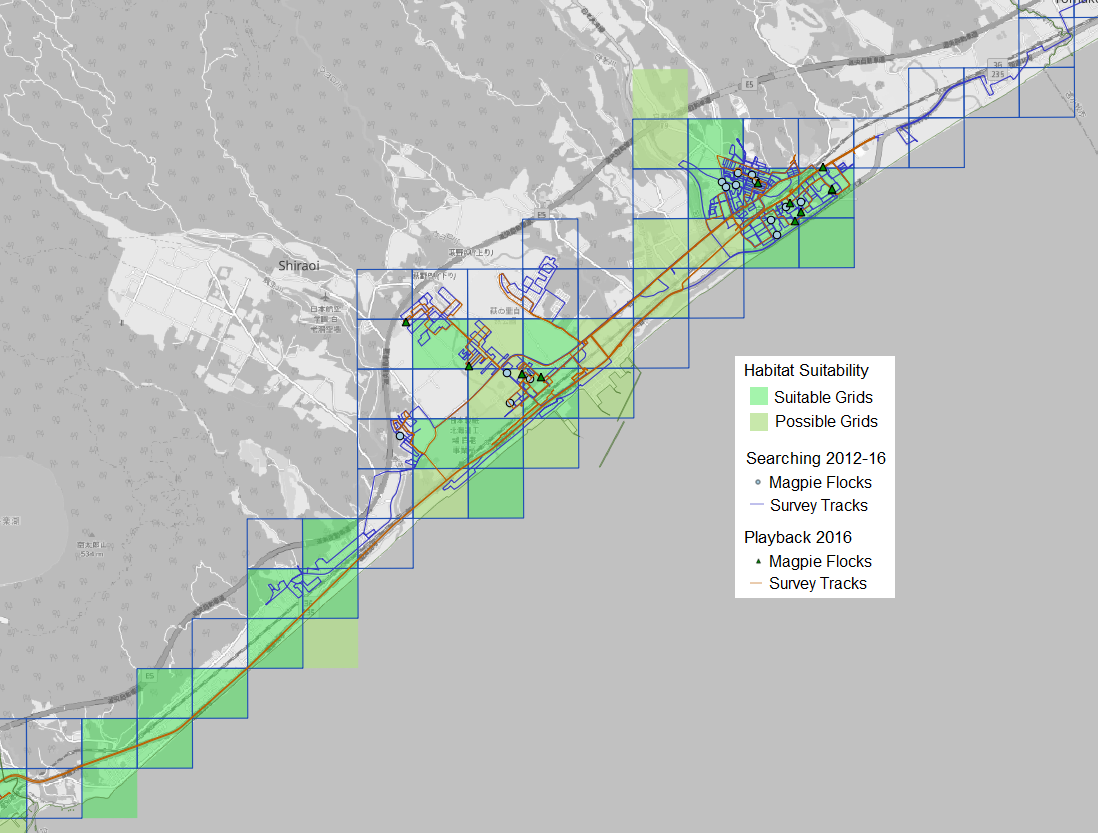

Supplement: Supplementary file 1 [file vetsci-13-00628-s001.zip › D8_Shiraoi.png]

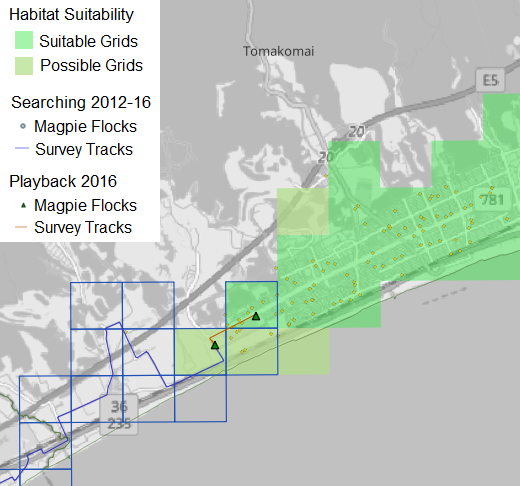

Supplement: Supplementary file 1 [file vetsci-13-00628-s001.zip › D9a_Tomakomai-west.png]

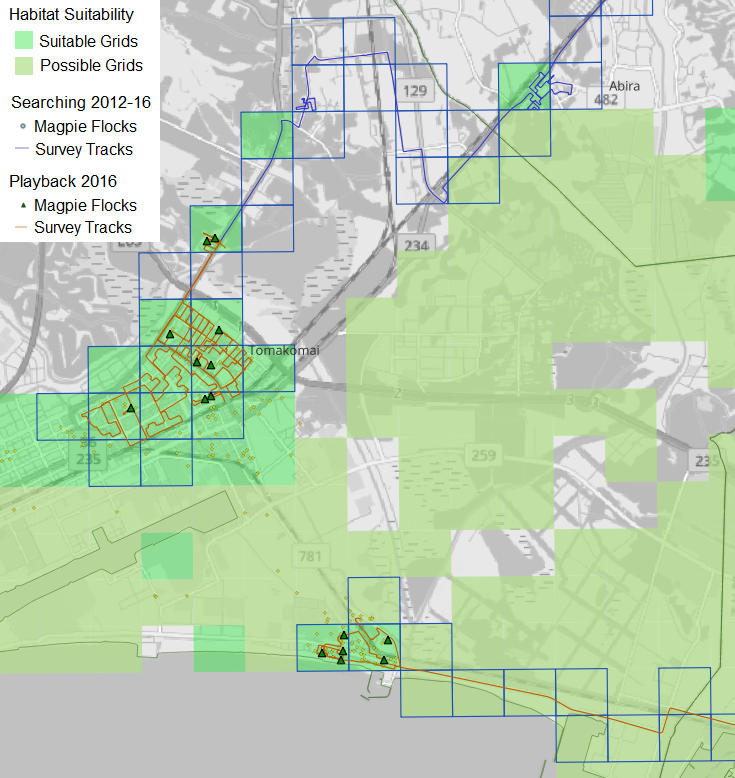

Supplement: Supplementary file 1 [file vetsci-13-00628-s001.zip › D9b_Tomakomai-east.png]
